# Supplementary material for: Chromothripsis during telomere crisis is independent of NHEJ, and consistent with a replicative origin
Source: Genome Res. 2019 May;29(5):737–49. doi: 10.1101/gr.240705.118 (PMC6499312; doi:10.1101/gr.240705.118)
Supplement: Supplemental Material [file supp_gr.240705.118_Supplemental_file_1.zip › contigs/annotated_contigs/DB116/contig.2.DB116_length_309_mean_cov_5.82524271845.docx]

**DB116_length_309_mean_cov_5.82524271845**

```
GCTAGAGAGGCTACTATCAGGAAGTGAAATGGATCATTTCTAATGGTAGGTCTCCATTGCCTACATGGTATTTCTGAGAGTGGTATCAG
 >chr3:21457820-21458066 - E=5e-134 p=7e-03
GGTGTCCAGTGAAAGATGGATAGGTTGACATGTCATGCTCATTTGGCCTAAAGGTTGTTACTGCCAGGAATGGACTTACAACTTACCAG

AATGTGAATGGTGACAAAAGGCAGGTTCTACTCTGGAAAAATATGTGTTATTTTATT|AGAAGTTCATG|CAGGTCATGAAGAGTCACA
                                                          >chr3:21457434-21457508 - E=3
GAGTTTAAAGAATTCTCTGGTTGGGTCATCAATGTTCAGATGTCe-33
```
